# Diagnostic accuracy of combined pulp sensibility tests for detecting post-anesthetic pulpal anesthesia in mandibular molars with symptomatic irreversible pulpitis

**DOI:** 10.1038/s41598-026-49432-z

**Published:** 2026-05-06

**Authors:** Tamanna Arora, Rakesh Singla, Gurdeep Singh Gill, Namita Jain, Arindam Sarkar, Adel Saeed N. Alobaid, Suraj Arora, Shan Sainudeen, Ahmed Babiker Mohamed Ali, Anshad M. Abdulla, Priyanka Saluja, Waled Abdulmalek Alanesi, Gotam Das

**Affiliations:** 1 Department of Conservative Dentistry and Endodontics, Jan Nayak Chaudhary Devilal Dental College, Sirsa, Haryana India; 2 Department of Oral Medicine and Radiology, Jan Nayak Chaudhary Devilal Dental College, Sirsa, Haryana India; 3 Department of Restorative Dental Sciences, College of Dentistry, King Khalid University, 61421 Abha, Saudi Arabia; 4 Department of Pediatric Dentistry & Orthodontic Sciences, College of Dentistry, King Khalid University, 61421 Abha, Saudi Arabia; 5 Mike Petryk School of Dentistry, University of Alberta, Edmonton, AB T6G1C9 Canada; 6 Department of Operative Dentistry, Faculty of Dentistry, University of Science and Technology, Inmaa City, Aden Yemen; 7 Department of Prosthodontics, College of Dentistry, King Khalid University, 61421 Abha, Saudi Arabia

**Keywords:** Heat test, Pulpal anesthesia, Pulp sensibility test, Symptomatic irreversible pulpitis, Diseases, Health care, Medical research

## Abstract

Attaining pulpal anesthesia (PA) is the key to painless endodontic treatment. Few studies have measured post-anesthetic PA using cold and/or electric stimuli but heat test has never been used for such purpose. Our study employed different combinations of Heat Test (HT), Cold Test (CT) and electrical pulp test (EPT) to detect post-anesthetic PA in mandibular molars with Symptomatic Irreversible Pulpitis. One hundred healthy endodontic patients received Inferior Alveolar Nerve Block with 2% lignocaine containing 1:200000 adrenaline. After achieving lip numbness, PA was evaluated using EPT, CT and HT. Negative response to PSTs indicated attainment of PA. Teeth exhibiting negative response to at least 2 PSTs were subjected to endodontic intervention. Outcomes of combinations of PSTs were compared with pain during endodontic treatment (gold standard test) using Fisher–Freeman–Halton exact test. No/mild pain (Heft Parker visual analogue scale) during endodontic treatment was considered as anesthetic success. Eighty-three patients completed the study and were statistically analysed. An overall significant association was found between PST combinations and the incidence of intraoperative pain (*P* = 0.004). The triple combination (EPT, CT and HT) showed the lowest risk of moderate/severe pain at 14.3% (95% CI 7.6–25.3). In individual test analysis, only Heat Test showed a statistically significant association with intraoperative pain (*P* = 0.001). This highlights the importance of using all 3 PSTs in detecting the presence of PA. *Clinical trial registration*: https://ctri.nic.in/, Registration number: CTRI/2023/03/051049, Registration date: 24 March.

**Supplementary Information:**

The online version contains supplementary material available at 10.1038/s41598-026-49432-z.

## Introduction

In endodontic practice, anesthetic success is defined as no or minimal pain during root canal treatment. Establishing pulpal anesthesia (PA) is fundamental to achieving anesthetic success because endodontic procedures involve direct manipulation of the dental pulp, a tissue that is richly innervated and highly sensitive to noxious stimuli. In cases of symptomatic irreversible pulpitis (SIP), pulpal nociceptive fibers become sensitized by inflammatory mediators, resulting in heightened pain perception and reduced anesthetic efficacy^1^. Adequate PA ensures effective blockade of these nociceptive pathways before instrumentation of the pulp chamber and root canals. Failure to achieve profound PA may result in intraoperative pain, increased patient anxiety, interruption of treatment, and reduced patient cooperation. Moreover, repeated painful stimuli may contribute to central sensitization, lowering the pain threshold and further complicating anesthetic management. Therefore, reliable establishment and verification of PA are essential prerequisites for successful and atraumatic endodontic therapy.

The inferior alveolar nerve block (IANB) is the most commonly used anesthetic technique to achieve PA in mandibular teeth undergoing endodontic treatment. Traditionally, the presence of lower lip numbness following IANB has been regarded as the primary clinical indicator of achieving PA and the point at which endodontic access is initiated. However, evidence indicates that despite achieving subjective lip anesthesia, many patients still experience pain during endodontic procedures, suggesting that lip numbness alone is not a dependable indicator of profound PA^2,3^.

To address this limitation, different studies have employed pulp sensibility tests (PSTs), such as the Electric Pulp Test (EPT), Cold Test (CT), or their combination, to objectively evaluate post-anesthetic pulpal status^4–15^. In these studies, a negative response to cold and/or electric stimulation was interpreted as evidence of successful PA. However, clinical findings have demonstrated discrepancies between PST results and the actual intraoperative pain experience. For instance, studies utilizing EPT have reported that approximately 16%–33% of patients who exhibited no response to electrical stimulation experienced pain during endodontic access preparation^4,8,11,14^. Similarly, investigations using CT have shown that 8.2%–62% of patients with no response to cold stimulation reported pain during pulp manipulation^5–7,9,10,13^. Furthermore, studies employing combinations of EPT and CT demonstrated that 23%–39% of patients experienced intraoperative pain despite negative responses to both stimuli^12,15^. These findings indicate that the absence of response to electric and/or cold stimuli does not necessarily correspond to adequate PA and cannot reliably predict pain-free endodontic treatment.

The dental pulp is predominantly innervated by unmyelinated C fibers and myelinated Aδ fibers. C fibers are centrally located within the pulp and constitute approximately 70–80% of the total pulpal nerve supply, whereas Aδ fibers are distributed more peripherally and account for 10–20% of pulpal nerve fibers^16^. Aδ fibers have a low activation threshold and are readily stimulated by thermal (cold or heat) or electric stimuli, whereas C fibers possess a higher activation threshold and are generally not activated by electric or cold testing^16–18^.

Considering the predominance of C fibers in the pulpal nerve supply, assessment of their activity alongside Aδ-fiber evaluation may provide a more reliable and clinically relevant indicator of adequate PA. Evidence suggests that C fibers are more likely to be activated by heat stimulation^16^. In inflamed pulps, application of heat may increase intrapulpal pressure, potentially stimulating both C fibers and Aδ fibers and thereby eliciting a pain response^16^.

Theoretically, integrating HT along with EPT and CT may provide a more comprehensive assessment of pulpal nerve activity and improve the prediction of anesthetic adequacy. Establishing a more reliable method to confirm profound PA before initiating endodontic access could reduce the risk of intraoperative pain, enhance patient comfort, and improve the overall predictability of endodontic treatment.

Therefore, the present study aimed to evaluate the diagnostic performance of different combinations of HT, CT, and EPT in assessing post-anesthetic pulpal anesthesia in mandibular molars with SIP. In addition, the study sought to determine whether these combinations could predict the occurrence of intraoperative pain during endodontic treatment, using the patient’s pain experience during the procedure as the gold standard. The null hypothesis of the present study was that different combinations of PSTs would not accurately detect adequate PA or predict the occurrence of intraoperative pain during endodontic treatment.

## Methods

**Fig. 1:**
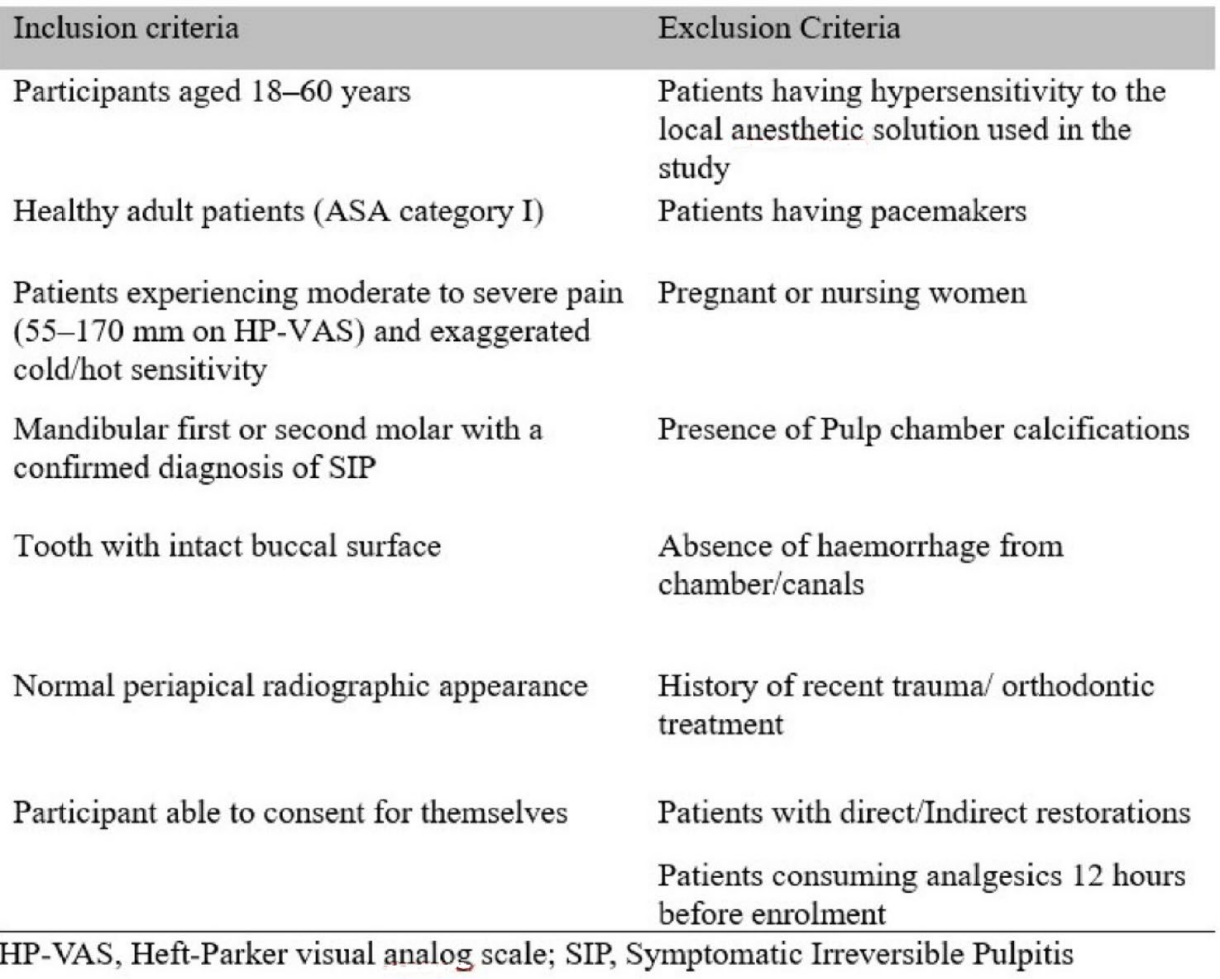
Inclusion and exclusion criteria.

This prospective single blind diagnostic study was designed and reported in accordance with the Standards for Reporting of Diagnostic Accuracy Studies (STARD) 2015 guidelines. After approval by the Institutional Ethical Committee of J.C.D Dental College, Sirsa, India (approval code: JCDV/DC/22/948, date of approval: 10-06-2022), the study was prospectively registered in https://ctri.nic.in under the registration number CTRI/2023/03/051,049 on 24 March 2023. The study was conducted from May 2023 to May 2024 in accordance with relevant guidelines and regulations. Written informed consent was obtained from each patient. Participants were randomly selected from the OPD of the Department of Conservative Dentistry and Endodontics, according to inclusion and exclusion criteria (Fig. 1). The target condition was defined as SIP in mandibular molars with confirmed pulpal vitality. The presence of bleeding from pulp chamber and root canals was considered definitive evidence of vital pulp tissue. Teeth that did not exhibit bleeding were excluded to ensure that anesthetic efficacy was evaluated exclusively in teeth with vital, inflamed pulpal tissue, thereby minimizing misclassification bias.

### Study design

All patients received standard IANB with 1.8 mL 2% lignocaine containing 1:200,000 adrenaline (Cadila Pharmaceuticals, India). In patients reporting lip numbness, PA was evaluated using EPT (Digitest; Parkell Inc, USA), CT (Endo Frost; Coltene) and HT (Sure-Endo temporary stopping, Korea) in a randomised manner. If lip numbness was not reported within 15 min of local anesthesia (LA), the patient was excluded from the study. All PSTs were performed by a single consultant endodontist under cotton roll isolation, and the stimulus was applied on the middle third of the buccal surface of the involved molar. All PSTs were conducted on a healthy contralateral tooth prior to testing the tooth in question.

To perform EPT, a lip clip was placed on the electrolyte-coated lip on the opposite side of the involved molar, making good contact with the mucosa. After that, an electrolyte (toothpaste) coated probe tip was placed on the molar and current was automatically increased at a medium rate (from 0 to 64) until patient felt tingling/pain^19^.

For CT, a no. 2 sterile cotton pellet saturated with Endo frost (− 50 °C) was placed on the test site for a maximum of 18 s or until the participant felt a sensation^19^.

For HT, petroleum jelly was applied to the test site to prevent the gutta-percha (GP) from sticking to the enamel surface. The GP stick was heated on a gas flame until it just began to smoke or slump and was then carefully placed onto the test tooth for 18 s or until the participant felt sensation^19^.

To ensure uniform temperature in all patients (− 50 °C for CT and + 80 °C for HT), a digital infrared thermometer (Microtek, India) was used. The temperature was recorded at the tip of the heated gutta-percha or cotton pellet immediately before application to the tooth surface to ensure a consistent stimulus of approximately + 80 °C or − 50 °C respectively. The temperature was verified prior to each application to maintain reproducibility of the thermal stimuli throughout the study. A time interval of 5 min was designated between each PSTs^19^.

The outcome of each PST was dichotomized as either positive or negative. A positive response was defined as the presence of pain, discomfort, or any perceptible sensation during or after application of the stimulus and was interpreted as indicating non-attainment of PA.

A negative response, defined as complete absence of pain or sensation during and after stimulation, was considered suggestive of attainment of PA.

In this diagnostic accuracy study, EPT, CT and HT were considered index tests. The adequacy of PA was evaluated using different combinations of these index tests. Participants who demonstrated negative responses to at least 2 of the 3 PSTs were considered adequately anesthetised and were subjected to endodontic treatment.

In patients exhibiting positive response to at least 2 (or all 3) PSTs, supplemental injections were administered using the same anesthetic solution and PSTs were repeated after 5 min. If the desired outcome (negative response to 2 PSTs) was not obtained, the patient was further retorted to the next method of supplemental anesthesia. The following sequence was observed for supplemental anesthesia: Buccal infiltration (1 ml), intra-ligamentary injection (0.2 ml per root), and repeat IANB (1.8 ml). Based on the predefined criterion (≥ 2 negative responses), four index test combinations were analyzed:

1. All three tests negative (EPT + CT + HT)
2. EPT and CT negative
3. EPT and HT negative
4. CT and HT negative

Endodontic procedure: A single experienced operator initiated endodontic access under rubber dam isolation (GDC, India) using an Endo Access Kit (Dentsply Sirona, PA). Canal orifices were located with DG 16 Endodontic Explorer (GDC, India). Stainless steel K-files #06, 08, and 10 (Mani, Japan) were used for the initial instrumentation of the root canals. If patients experienced pain anytime during the endodontic procedure, the procedure was immediately stopped, and patients rated their pain using Heft-Parker Visual Analogue Scale (HP-VAS)^20^. This is a 170-mm scale divided into 4 categories: no pain (0 mm), mild pain (1–54 mm), moderate pain (55–114 mm), and severe pain (115–170 mm).

The reference standard for determining the true pulpal anesthetic status was the presence or absence of pain during endodontic treatment. No or mild intraoperative pain (0–54 mm) indicated anesthetic success whereas moderate to severe pain (55–170 mm) experienced during the procedure indicated anesthetic failure. The diagnostic performance of PSTs was evaluated by comparing the index test results with the reference standard (intraoperative pain). The categories were defined as follows:

- **True negative (TN)** A negative PST response followed by no or mild intraoperative pain, indicating correctly predicted presence of PA.
- **True positive (TP)** A positive PST response followed by moderate to severe pain during the endodontic procedure, indicating correctly predicted absence of PA.
- **False negative (FN)** A negative PST response followed by moderate to severe intraoperative pain, indicating failure of the index test to detect inadequate PA.
- **False positive (FP)** A positive PST response followed by no or mild pain during the procedure, indicating incorrect prediction of adequate PA.

For each PST, sensitivity (SN), specificity (SP), positive predictive value (PPV), negative predictive value (NPV), and accuracy (AC) with 95% confidence intervals were calculated using MedCalc’s online diagnostic test statistical calculator.

- **Sensitivity (SN)** The ability of a test to identify teeth with actual anesthetic failure.
- **Specificity (SP)** The ability of a test to identify teeth with actual anesthetic success.
- **Positive predictive value (PPV)** The percentage of subjects with a positive response to the test who truly had anesthetic failure.
- **Negative predictive value (NPV)** The percentage of subjects with a negative response to the test who truly had anesthetic success.
- **Accuracy (AC)** The proportion of true results (both true positives and true negatives) among the total number of cases examined.

### Sample size calculation

The study’s sample size was calculated using n Master software version 2 (Hitech Private Limited Coimbatore, India), based on a previous study^19^. Anticipating 100% sensitivity of the reference test, with a confidence interval of 95% and power of 90%, the minimum sample size of the study turned out to be 80.

### Blinding and randomization

The sequence of PSTs was decided based on a randomly chosen envelope containing the names of all 3 PSTs in a different order. A single consultant endodontist administered local anesthesia/supplemental injections and performed all PSTs. After obtaining negative responses from at least 2 PSTs, the patient was handed over to the operator for endodontic treatment. In this manner, the consultant was blinded to the final study outcome, and the operator was unaware of supplementary injections and responses of PSTs.

### Statistical analysis

Descriptive statistics were expressed as mean ± standard deviation for continuous variables and frequency with percentage for categorical variables. Independent t-test was used for age comparison and Chi-square test was used for gender and type of teeth. Logistic regression analysis was done to detect association between variable (gender and tooth type) and intraoperative pain. To evaluate the association between different PST combinations and risk of intraoperative pain, an omnibus Fisher–Freeman–Halton exact test was performed followed by Post-hoc pairwise Fisher’s exact tests.

To assess the diagnostic performance of individual PST with respect to intraoperative pain, Fisher’s Exact Test was used. A *p*-value < 0.05 was considered statistically significant. All statistical analysis was performed using SPSS software program version 21 (IBM, USA).

## Results

**Fig. 2:**
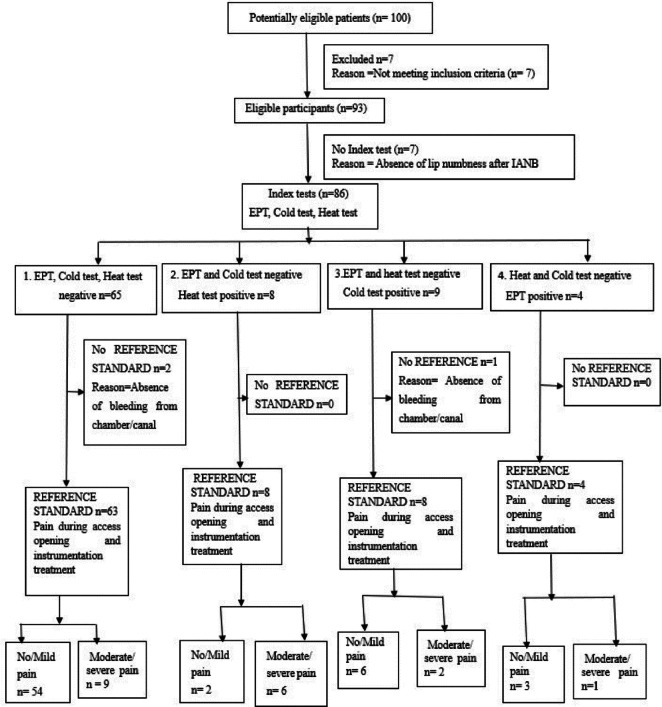
Flow diagram of the study.

One hundred patients were examined for the study and after applying inclusion/exclusion criteria, 83 patients completed the study and were statistically analysed (Fig. 2). To achieve a negative response to 2 or 3 PSTs, additional injections/blocks were administered in 40 patients (26 required buccal infiltration, 9 required an additional intraligamentary injection, and 5 required a repeat IANB). Overall, 65 patients (78.3%) reported no/mild pain, while 18 patients (21.7%) experienced moderate to severe pain during endodontic treatment. Throughout the course of the study, no adverse events or complications were encountered.

Demographically (Table 1), a significant difference was found in gender (Males > Females, *p* = 0.02) and tooth type (1st molars > 2nd molars, *p* = 0.04). Logistic regression analysis (Table 2) revealed that tooth type was a significant predictor of intraoperative pain (*p* = 0.03). In contrast, gender was not significantly associated with intraoperative pain (*p* = 0.99). Table 3 presents the association between different PST combinations and intraoperative pain. The most frequent combination was the simultaneous use of EPT, CT and HT**,** observed in 63 patients, out of which 54 patients (85.7%) experienced no or mild pain**,** whereas 9 patients (14.3%) reported moderate to severe pain, corresponding to a risk of moderate/severe pain of 14.3%. A significant association was observed between the PST combinations and intraoperative pain (Fisher–Freeman–Halton exact test, *P* = 0.004). Post-hoc pairwise comparisons (Fisher’s Exact Test) demonstrated that the EPT + CT + HT combination resulted in a significantly lower incidence of moderate/severe pain compared with the EPT + CT combination, whereas no other pairwise differences were statistically significant. When individual tests were analysed using Fisher’s Exact Test (Table 4), the HT showed a significant association with intraoperative pain (*P* = 0.001), whereas EPT and CT were not significantly associated with intraoperative pain (*P* = 0.869 and *P* = 0.811, respectively). Performance measure values for individual PSTs like SN, SP, PPV and NPV and AC are given in Table 5.

**Table 1:** Demographic Table- Gender, Age and tooth type.

Gender	n (%)	Age (Mean ± SD)	1st molar n (%)	2nd molar n (%)
Male	52(62.7%)	32.36 ± 10.97	27(52.9%)	25(78.1%)
Female	31(37.3%)	33.35 ± 10.77	24(47.1%)	7(21.9%)
Overall	83	32.73 ± 10.84	51(61.4%)	32(38.6%)
*P* value	0.02*	0.69	0.04*	

*P value (*p* < 0.05) significant.

**Table 2:** Association between gender and tooth type with pain during endodontic treatment.

Variable		n (%)	No/Mild pain n (%)	Moderate/ severe Pain n (%)	*P* value
Gender	Males	52(62.6%)	41 (78.8%)	11 (21.2%)	0.99, NS
	Females	31(37.3%)	24 (77.4%)	7 (22.6%)	
Tooth	First molar	51(61.4%)	44 (86.3%)	7 (13.7%)	0.03, S
	Second molar	32(38.5%)	21 (65.6%)	11 (34.4%)	

S, significant; NS, non-significant.

*P* value (*p* < 0.05) significant.

**Table 3:** Association between PST Combination and Intraoperative Pain (Fisher–Freeman–Halton exact test).

PST combination (outcome negative)	Total n (%)	No/mild pain n (%)	Moderate/severe pain n (%)	Risk of moderate/severe pain % (95% CI)
EPT + CT + HT^a^	63 (76.0)	54 (85.7)	9 (14.3)	14.3% (7.6 – 25.3)
EPT + CT^b^	8 (9.6)	2 (25.0)	6 (75.0)	75.0% (40.9 – 92.9)
EPT + HT^ab^	8 (9.6)	6 (75.0)	2 (25.0)	25.0% (7.1 – 59.1)
CT + HT^ab^	4 (4.8)	3 (75.0)	1 (25.0)	25.0% (4.6 – 69.9)
Total	83 (100)	65 (78.3)	18 (21.7)	

Overall association: Significant (*P* = 0.004).

Groups with different superscript letters differ significantly in the risk of moderate/severe intraoperative pain.

**Table 4:** Comparison of Individual PST Outcomes and Intraoperative Pain (Fisher’s Exact Test).

Test	Test outcome	Total n (%)	No/mild pain n (%)	Moderate/severe pain n (%)	Risk of moderate/severe pain % (95% CI)	*P* value*
EPT	Negative	79 (95.1)	62 (78.5)	17 (21.5)	21.5% (14.0–31.6)	0.869
	Positive	4 (4.8)	3 (75.0)	1 (25.0)	25.0% (4.6–69.9)	
Cold Test	Negative	75 (90.4)	59 (78.7)	16 (21.3)	21.3% (13.6–31.7)	0.811
	Positive	8 (9.6)	6 (75.0)	2 (25.0)	25.0% (7.1–59.1)	
Heat Test	Negative	75 (90.4)	63 (84.0)	12 (16.0)	16.0% (9.4–26.0)	**0.001**
	Positive	8 (9.6)	2 (25.0)	6 (75.0)	75.0% (40.9–92.9)	
Total		83 (100)	65 (78.3)	18 (21.7)

**Table 5:** Performance measure values for individual pulp sensibility tests.

Term	EPT (95% CI)	COLD TEST (95% CI)	HEAT TEST (95% CI)
Sensitivity	5.6% (0.1–27.3%)	11.1% (1.4–34.7%)	33.3% (13.3–59.0%)
Specificity	95.4% (87.1–99.0%)	90.8% (81.0–96.5%)	96.9% (89.3–99.6%)
PPV	25.0% (3.6–75.1%)	25.0% (6.8–60.2%)	75.0% (39.8–93.1%)
NPV	78.5% (76.3–80.4%)	78.7% (75.5%–81.5%)	84.0% (79.0–87.9%)
Accuracy	75.9% (65.2–84.6%)	73.5% (62.6–82.6%)	83.1% (73.3–90.4%)

## Discussion

Achieving profound PA is a fundamental prerequisite for painless endodontic treatment. In clinical practice, the adequacy of PA is ultimately confirmed during the operative procedure itself, particularly at the stage of access cavity preparation. The occurrence of pain at this stage indicates inadequate anesthesia and necessitates the immediate administration of supplemental anesthetic techniques. Clinicians often use supplementary techniques to achieve adequate PA (or enhance anesthetic success), but once patient experiences pain during root canal treatment, it compounds anxiety and breeds distrust in patient’s mind. Therefore, reliable preoperative methods to verify the adequacy of PA are essential to minimize the risk of intraoperative pain and ensure patient comfort during endodontic procedures.

The current study employed different possible combinations of HT, CT and EPT to evaluate post anesthetic PA (or risk of intraoperative pain) with a null hypothesis that different combinations would not be significantly associated with reduced likelihood of intraoperative pain.

Results from our study demonstrated that a negative outcome to the 3 PST combination resulted in a significantly reduced risk of moderate to severe intraoperative pain. The null hypothesis was therefore not accepted.

The observed findings may be explained by the physiological basis of pulp sensibility testing. In teeth with irreversible pulpitis, unclarified multifarious neuroinflammatory and neuropulpal interactions take place^21^. During pulpal inflammation, intrapulpal pressure changes profoundly affect sensory nerves of differing diameters, with the increase in pressure selectively blocking larger diameter A-delta fibers and activating the smaller C-fibers^22^. As a result, pulpal nerve fibers may exhibit altered responsiveness, which may lead to inconsistent responses to individual pulp sensibility tests. The substantially lower incidence of intraoperative pain observed with the triple-test combination suggests that multimodal stimulation of pulpal nerve fibers may improve the detection of residual pulpal responsiveness prior to access cavity preparation.

Compared to the triple test combination, the dual test combinations showed a greater tendency toward intraoperative pain. These findings suggest that reliance on a limited number of PSTs may increase the likelihood of incomplete anesthesia verification. However, these observations are exploratory and should be interpreted cautiously because the number of observations in the dual-test groups was relatively small compared with the triple-test group, which may have reduced the precision of the risk estimates. Further studies with larger and more balanced sample sizes are therefore necessary to confirm these trends.

The present study involved both diagnostic and intervention sections. In the diagnostic part of the study, PA was assessed by using different combinations of PSTs. In the intervention part, endodontic treatment was done, which was guided by the outcome of combined PSTs. Therefore, this study can also be considered as a test guided clinical decision strategy. However, since the prime objective of the study was to diagnose PA; it was labelled as diagnostic trial, and hence the STARD guidelines were followed.

Although the primary objective of the present study was to evaluate PA using combinations of PSTs, the independent application of each test also allowed assessment of their individual diagnostic performance. As EPT, CT and HT employ distinct stimuli, their results can be interpreted separately to examine their association with intraoperative pain. The analysis revealed that neither EPT nor CT showed a statistically significant association with intraoperative pain (*p* = 0.869 and 0.811, respectively), with comparable risks of moderate to severe pain observed in both negative and positive test outcomes. This suggests that these tests, when used individually, have limited predictive value for confirming PA. In contrast, HT demonstrated a statistically significant association (*p* = 0.001), with a markedly higher incidence of moderate to severe pain in teeth exhibiting a positive response (75%) compared to those with a negative response (16%). This finding indicates that a positive heat test may be a more reliable indicator of inadequate pulpal anesthesia.

The diagnostic performance measures further support these observations. All PSTs demonstrated high specificity (> 90%), indicating a strong ability to identify cases without intraoperative pain. However, sensitivity was low across all tests, particularly for EPT (5.56%) and CT (11.11%), reflecting their poor ability to detect patients who would experience pain during treatment. The HT showed comparatively higher sensitivity (33.33%) and a substantially higher PPV (75%), suggesting better clinical utility when the test response is positive. Nevertheless, the NPVs of all tests were only moderate (approximately 78–84%), indicating that a negative response does not reliably exclude the possibility of intraoperative pain. Importantly, even among cases with negative responses, a notable proportion of patients continued to experience pain, highlighting the inherent limitations of relying on any single PST.

HT is not routinely employed in endodontic/diagnostic studies because of several limitations. First, there are safety concerns regarding pulp and surrounding soft tissues; second, it is challenging to provide a consistent heat stimulus; and lastly, there may be delay in eliciting a response ^18,23,24^. In our study however, safety to pulp was not a concern as pulpectomy was scheduled. Further, heated GP stick was judiciously applied on the test site, ensuring no contact with the gingival tissues. Every time, a new GP stick was used for each test to ensure that no heat was retained and the stick’s shape wasn’t distorted, thus providing consistent contact with the tooth. HT can be performed by various methods such as hot water baths, frictional heat, heated GP or compound stick or electronic heat testing instruments. We acknowledge that using GP stick might be a limitation as it is difficult to control the temperature obtained by heating gutta-percha over a flame ^18,21^. To address this, a digital infrared thermometer was used to ensure uniform temperature across all patients. While using HT, there may be delay in eliciting a response^18,25^. Therefore, after applying heat, a waiting period of 10 s was employed to allow for the onset of symptoms^25^. Furthermore, we allowed a time interval of 5 min between each PST to enable the pulp to return to its normal temperature^19^. Performing all three PSTs in every patient (including time interval) resulted in increased chairside time. In addition, the need for supplemental injections in a substantial proportion of cases led to higher cumulative anesthetic dosage, with a potential risk of injection-site trauma. It is important to note that this sequential testing protocol, including defined recovery intervals, was implemented under controlled university clinical settings to ensure methodological standardization and may not be directly feasible in routine chair-side practice.

A single consultant endodontist performed all PSTs to prevent operator associated variability^26^. Only systemically healthy patients were included as pulp response can be altered in diseased individuals^27,28^. Ideally, PSTs should be performed under rubber dam isolation; however, due to the study design (which involved repeated supplementary injections), cotton roll isolation was performed. We excluded patients who consumed analgesics within 12 h of enrolment, as they might affect the pulpal response to the cold sensibility test^29^. After IANB, one patient who failed to report lip numbness was excluded from the study since the lack of soft-tissue anesthesia indicates a missed block^30^. In our study, 3 patients were excluded due to the absence of bleeding upon access. Although this was necessary to verify the target condition of vital SIP, this may introduce spectrum bias. Consequently, the diagnostic accuracy of PSTs reported in the current study applies specifically to vital teeth and may not be generalisable to teeth with partial pulpal necrosis.

The present study utilized 1.8 mL of anesthetic solution for the initial injection. Based on the predefined diagnostic protocol, 40 patients who did not exhibit a negative response to at least two index tests were administered supplemental injections. As a result, the PSTs were not functioning as independent predictors of the reference standard (intraoperative pain), but rather were incorporated within a test–treatment algorithm. This methodological approach may have led to an overestimation of specificity and negative predictive value, while also constraining the distribution of test positivity at the clinical decision time-point.

Furthermore, these 40 patients received a higher total volume of anesthetic solution compared to others. Since higher volumes of anesthetic solutions are associated with increased anesthetic success,^31^ this could be considered a potential confounding factor. However, it is important to emphasize that the primary objective of the present study was to evaluate the ability of PSTs to detect PA, rather than to enhance anesthetic success. Furthermore, as endodontic access was limited to patients demonstrating negative responses to at least 2 PSTs, the findings should be interpreted in the context of potential verification bias. However, this approach was ethically justified to minimize the risk of subjecting patients to painful procedures in the presence of inadequate anesthesia.

One notable finding of our study was that despite negative responses to all 3 PSTs, approximately 14% of patients still experienced intraoperative pain. This finding suggests that sensibility testing alone may not always completely predict anesthetic success. Factors such as individual variability in pain perception or preoperative patient anxiety, which were not evaluated in the present study, may also influence intraoperative pain experience. Therefore, the 3 PSTs approach should be regarded as a risk-stratification adjunct rather than a substitute for intraoperative pain assessment during endodontic procedures.

Future studies incorporating psychological assessment parameters, larger sample sizes with diverse pulpal diagnoses, the use of electronic heat devices, and additional objective methods for verifying pulpal anesthesia may provide deeper insight into this observation.

## Conclusion

Within the limitations of the present study, negative responses to electric pulp, cold, and heat tests were associated with a lower likelihood of intraoperative pain but did not ensure complete pulpal anesthesia. The persistence of moderate to severe pain in 14% of patients despite negative test responses highlights the inherent limitations of pulp sensibility testing. Accordingly, these tests should be regarded as adjunctive tools for risk assessment and probabilistic indicators rather than definitive indicators of anesthetic success.

## Supplementary Information

Below is the link to the electronic supplementary material.

Supplementary Material 1

## Data Availability

All data supporting the findings of this study are available within the paper and its supplement information.
